# Efficacy of alemtuzumab and natalizumab in the treatment of different stages of multiple sclerosis patients

**DOI:** 10.1097/MD.0000000000009908

**Published:** 2018-02-23

**Authors:** Li Wang, Chun-Hui Qi, Ren Zhong, Chao Yuan, Qiu-Yue Zhong

**Affiliations:** a Department of Pharmacy, Jining No.1 People's Hospital, Jining; b Department of Pharmacy, Weifang People's Hospital, Weifang; c Department of Neurology, Zhucheng People's Hospital, Zhucheng, P.R. China.

**Keywords:** alemtuzumab, efficacy, multiple sclerosis, natalizumab

## Abstract

**Background:**

Multiple sclerosis (MS) is an autoimmune disease, in which the insulating covers of nerve cells in the brain and spinal cord are demyelinated. This study was conducted to compare the efficacy of alemtuzumab and natalizumab in the treatment of different stages of MS patients.

**Methods:**

A total of 585 patients diagnosed with MS and hospitalized were included and analyzed after which they were divided into the primary progressive MS A and B groups, the relapsing-remitting MS (RRMS) C and D groups, and the secondary progressive MS E and F groups. Patients in A, C, and E groups were administered alemtuzumab while those in B, D, and F groups were administered natalizumab for the treatment. The expanded disability status scale (EDSS) scores and the EDSS difference were calculated before and after treatment. The number of head magnetic resonance imaging enhanced lesions in the patients, recurrence time and recurrence rate were measured before and after treatment.

**Results:**

The EDSS score of the RRMS group was significantly lower than that of the primary progressive MS group and the secondary progressive MS group. After 12 months of treatment, the EDSS score of RRMS patients treated with natalizumab was significantly lower compared with the patients with alemtuzumab, and the difference before and after treatment was significantly higher than alemtuzumab. The recurrence rate of the RRMS-D group was significantly lower than the RRMS-C group. After 12 months of treatment, compared with the RRMS-C group, a significant reduction was observed in the number of head magnetic resonance imaging enhanced lesions and longer recurrence time in the RRMS-D group.

**Conclusion:**

The efficacy of natalizumab was better than alemtuzumab in the treatment of patients in the RRMS group, while there was no significant difference among other stages of MS patients, which provided the theoretical basis and clinical guidance for the treatment of different stages of MS.

## 1 Introduction

Multiple sclerosis (MS) is an autoimmune disease characterized by demyelination of white matter in the central nervous system.^[1]^ It usually affects white matter around the ventricle, optic nerve, spinal cord, brain stem and cerebellum, damaging the nerve axons, thereby consequently resulting in severe disability.^[2]^ The main predisposing factors lie in autoimmune impairment, viral infection, genetic predisposition, environmental factors, and it is characterized by accident attacks and neurologic damage.^[3]^ Nearly 2,500,000 people suffer from MS worldwide, and most of them are unable to keep on pace with their own daily lives due to the progression and development of the disease.^[4]^ In reality, 80% to 90% of MS patients encounter with a relapsing-remitting disease course in the beginning, along with different phases changing from deterioration, remission, and stability.^[5]^ However, it has been recorded that 10% to 20% of MS patients present with primary progressive course (PPMS) with constant deterioration, and with or without extra relapses from the onset of the disease.^[6]^ Clinically, the main diagnostic means for MS include detection of the neuroaxis by magnetic resonance imaging (MRI), analysis of patient's cerebrospinal fluid, and visual evoked potentials (VEPs).^[1]^ Previous studies found several treatments for relapsing-remitting multiple sclerosis (RRMS) in the last 20 years, but their relevant effects and adverse reactions still remain to be well understood, it is of great clinical importance for the neurologist to select a suitable treatment for different MS patients.^[7–10]^

With the rapid development and change in MS treatments, recently, FDA approved of 8 MS treatment regimens including glatiramer acetate, IFN-β-1a, IFN-β-1b, fingolimod, natalizumab, and alemtuzumab.^[11]^ Natalizumab, as a humanized monoclonal antibody, is a selective adhesion molecule inhibitor that prevents alpha4 integration and promotes the migration of peripheral blood lymphocytes to the central nervous system.^[12,13]^ Alemtuzumab is also a humanized monoclonal antibody, which functions on CD52 on the surface of T cells and B cells, which is currently used primarily as a treatment option for B-cell chronic lymphocytic leukemia and RRMS.^[14]^ By the depletion of pan lymphocytes and continuous modification of lymphocyte repertoire, alemtuzumab could lead to long-term disease stability for a majority of patients suffering from active disease.^[15]^ Based on this, in the current study, we intend to compare the efficacy of alemtuzumab and natalizumab in the treatment of different stages of MS patients and we hope it will provide certain clinical guidance for neurologists in the treatment of MS.

## 2 Materials and methods

### 2.1 Study subjects and grouping

Between January 2006 and October 2014, a total of 585 cases diagnosed with MS by cerebrospinal fluid examination, evoked potential and MRI, hospitalized in the department of neurology of Weifang People's Hospital and other hospitals were included in the study as subjects and were analyzed. The diagnosis was in accordance with the Poser Standard.^[16]^ The patients comprised of 254 males and 331 females and the age of onset was 13 to 76 years, the average age 40.68 ± 12.46 years and average duration of disease 33.01 ± 12.04 months. The inclusion criteria for the patients was as follows: patients with the corresponding clinical manifestations of MS; patients in line with MS diagnostic criteria; no limitation of age, sex, race, education level; patients with clinical symptoms, detailed signs and no other serious complications. Patients were excluded if they suffered from diseases capable of interfering with clinical studies, with severe depression or communication disorders. According to different treatment regimens, the patients were divided into 2 groups and according to the development of the disease, the patients were divided into 3 stages: the PPMS A and B groups, the RRMS C and D groups, and the secondary progressive MS (SPMS) E and F groups.^[17]^ Before treatment, data including the Expanded Disability Status Scale (EDSS), baseline characteristics, and history of diabetes mellitus or severe genetic diseases of patients were compared. EDSS, which is referred to as the gold standard for evaluating multiple sclerosis dysfunction currently, was employed as the most commonly employed clinical assessment scale for multiple sclerosis, and evaluation indicator widely used in clinical trials. Informal consents were retrieved from patients and their families for the experiment.

### 2.2 Treatment regimens

Based on the grouping, patients in the PPMS-A, RRMS-C, and SPMS-E groups were administered alemtuzumab at a dose of 12 mg/d for a course of 12 months as treatment. The patients were continuously administered intravenous injections on 5 days in the first month and 3 days in the twelfth month respectively. The patients in the PPMS-B, RRMS-D, and SPMS-F groups were given natalizumab for a course of 12 months, and they were administered 300 mg natalizumab via an intravenous injection every 4 weeks with continuous treatment and detailed observation and analysis.

### 2.3 Observation indicators

Kurtzke EDSS (with a range of 0–10 points)^[18]^ was employed to grade the nerve function on vertebral body, cerebellum, brainstem, sense, bladder, rectum, brain, and vision of MS patients before and after treatment. A score ranging between 0 to 3.0 points represented a mild disease; a score ranging between 3.0 and 5.5 points represented that patients could walk for a certain distance; a score ranging between 5.5 to 6.0 points indicated unilateral limb disorder; a score ranging between 6.0 to 7.0 points suggested that bilateral limbs needed help; a score ranging between 7.5 and 9.5 points represented an increase in the degree of disability and a need of someone else's daily external care; and a score of 10.0 points represented death of a patient. The number of patient's head MRI enhanced lesions was measured before and after treatment. The recurrence time was calculated and the EDSS score and recurrence rate of the patients in both groups were calculated.

### 2.4 Statistical analysis

SPSS19.0 statistical analysis software (IBM Corp, Armonk, NY) was employed to analyze the data, and the measurement data were expressed as mean ± standard deviation. The statistical analysis was performed by the *t* test. The counted data were presented as percentage or ratios. The *t* test was employed for comparing among the groups, and a difference of *P* < .05 was considered statistically significant.

## 3 Results

### 3.1 Baseline characteristics of patients in each group

Among the 585 MS patients, there were 254 males and 331 females. Their age of onset was 13 to 76 years with a mean age of 40.68 ± 12.46 years, in which patients between 20 to 40 years old represented a big population, with the average disease duration of 33.01 ± 12.04 months. According to the clinical classification, the patients were divided into 3 groups: the PPMS (n = 128), BMS (n = 344), and SPMS (n = 113). No significant differences were observed in terms of age, sex, and disease duration among the 3 groups (*P* > .05). The EDSS score of the BMS group was significantly lower than the score of the PPMS group and SPMS group (*P* < .05) (Table 1).

**Table 1:**
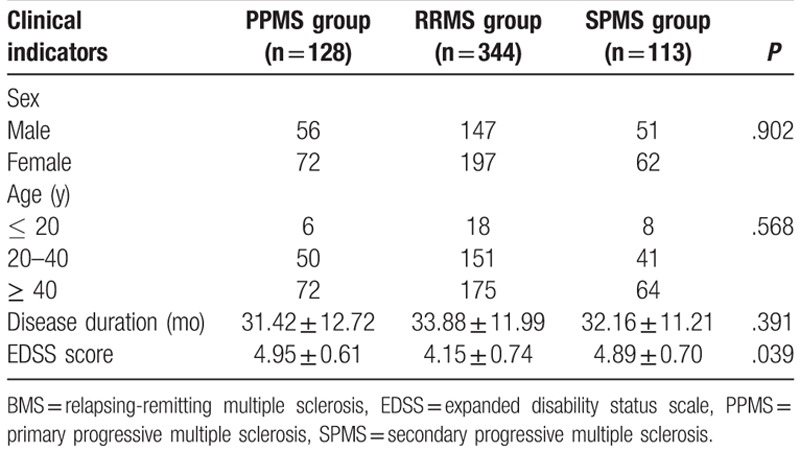
Baseline characteristics of patients in each group.

### 3.2 Patients treated with natalizumab express less adverse reactions than the alemtuzumab

Adverse reactions were divided into mild-moderate adverse reactions and severe adverse reaction. When the 2 treatment groups were compared, in terms of mild-moderate adverse reactions, the number of the patients who had any incidence of respiratory tract infection, headache, and nausea in the natalizumab treatment group was significantly lower than that in the alemtuzumab treatment group (*P* < .05). In terms of severe adverse reaction, in comparison with the alemtuzumab treatment group, the incidence of thyroid disease, immune platelet purpura, and liver function damage decreased in the natalizumab treatment group (*P* < .05). No significant differences were observed in the incidence of adverse reactions such as herpes infection, reduced blood lymphocytes, and urinary tract infections between the treatment groups (Table 2).

**Table 2:**
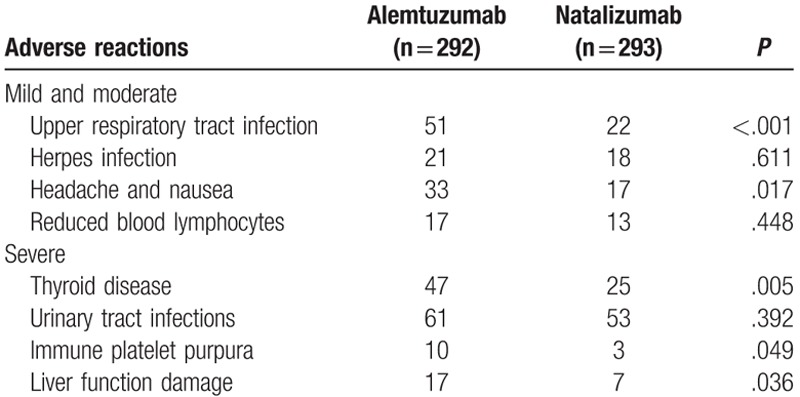
The natalizumab treatment groups have less adverse reactions than alemtuzumab treatment groups.

### 3.3 The effect of natalizumab was superior to alemtuzumab for the treatment of RRMS patients

In the 6 groups, patients in the PPMS-A group, the RRMS-C group, and the SPMS-E group were treated with alemtuzumab, while those in the PPMS-B group, the RRMS-D group, and the SPMS-F group were treated with natalizumab, and the course of duration was 12 months for all patients. As shown in Table 3, no significant differences were observed in terms of EDSS index and the difference value (D-value), between the PPMS and SPMS groups before and after treatment (*P* > .05). However, as for RRMS patients, the EDSS score in the natalizumab treatment group was significantly lower than the score in the alemtuzumab group, while the D-value was significantly higher before and after treatment in the natalizumab treatment group than the score in the alemtuzumab treatment group (*P* < .05), suggesting that for treating RRMS patients, natalizumab was superior to alemtuzumab.

**Table 3:**
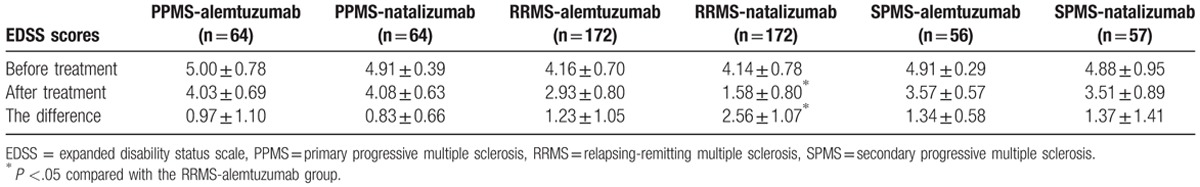
The natalizumab treatment groups shows lower EDSS score than that in the alemtuzumab groups.

### 3.4 The RRMS-D group has lower recurrence rate, more head MRI enhanced lesions and longer recurrence time than that in the RRMS-C group

As shown in Table 4, the recurrence rate of the RRMS-C group and the RRMS-D group was relatively low among the 6 groups, and the recurrence rate of the RRMS-D group was significantly lower than the RRMS-C group (*P* < .05). After 12 months of treatment, compared with the RRMS-C group, the RRMS-D group reduced the number of head MRI enhanced lesions (*P* < .05), but there was no statistical difference among other clinical types (*P* > .05). The recurrence time in the RRMS-D group was relatively longer than that in the RRMS-C group (*P* < .05), indicating that for treating RRMS patients, natalizumab was a more obvious option than alemtuzumab.

**Table 4:**
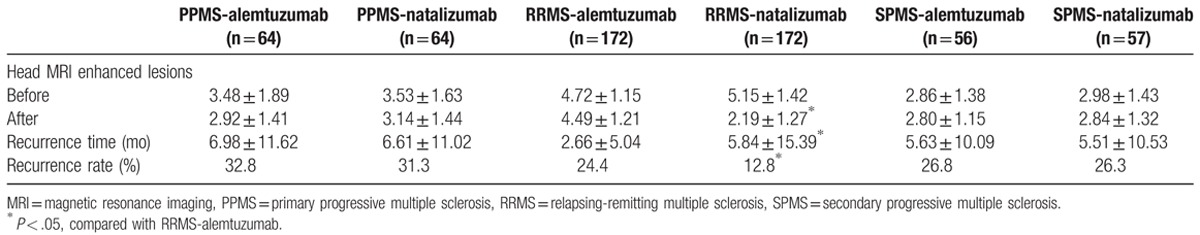
The RRMS-natalizumab group has more head MRI enhanced lesions than that in the RRMS-alemtuzumab group.

## 4 Discussion

MS, as a chronic inflammatory disorder, could disturb the normal order of the central nervous system, and is clinically represented by the progression of disability and relapse which may physically, socially, and psychologically influence the patients and bring a viscous financial burden to the patients and their families.^[19,20]^ A previous study illustrated the importance of a few suitable disease-modifying therapies for patients with MS, which could alleviate the course of the disease.^[21]^ Therefore, in this study, we compared the efficacy of alemtuzumab and natalizumab in the treatment of different stages of MS patients and we concluded that natalizumab was better than alemtuzumab for the treatment of RRMS patients without any significant difference among other stages of MS patients.

Our findings revealed that RRMS patients treated with natalizumab had significantly lower EDSS scores compared with the patients treated with alemtuzumab, which suggested that natalizumab was a superior option over alemtuzumab in the treatment of RRMS. The EDSS, as an ordinal but linear measurement, plays an important role in assessing the life quality of MS patients, and 1 published study illustrated that the improvement in the EDSS score after a relapse of 3 to 6 months and varied recovery from the relapse could increase the risk of RRMS disability, while the constant decrease even disappearance of relapses usually accompanies SPMS patients.^[22,23]^ Natalizumab, a type of monoclonal humanized antibody could bind and antagonize α4β1-integrin, which can effectively inhibit the migration of inflammatory cells through the blood–brain barrier.^[24]^ A prior study proved that natalizumab was universally considered the optimal treatment for RRMS.^[25]^ Alemtuzumab, also a humanized monoclonal antibody, could induce a lasting lymphopenia by supplementing the cell lysis of B and T cells,^[26]^ and previous data showed that alemtuzumab could impact the functional property of immune cells, which can promote the rebalance of the network responsible for the immune tolerance in MS.^[27]^ Polman et al^[28]^ demonstrated that the further progression of the disability evaluated by EDSS in MS patients treated with natalizumab had a reduction rate of 42% after 2 years compared with placebo, and its clinical relapse had reduced by 68% and patients’ head MRI enhanced lesions had decreased with a rate of 92%. Moreover, Farrel and Giovannoni^[29]^ showed that a dramatic relapse decrease was observed in MS patients treated with alemtuzumab along with, an increase in the EDSS score, and an increase in the number of MRI enhanced lesions in a patient's head. The data were in consistency with our findings suggesting that natalizumab was of higher efficacy than alemtuzumab in the treatment of MS.

Besides, in the current study, we found that natalizumab had lesser intensity of the adverse reactions than alemtuzumab in the treatment of MS, especially natalizumab had lower incidence of respiratory tract infection, headache, and nausea than alemtuzumab in terms of mild and moderate adverse reactions, and natalizumab had a significant decrease in thyroid disease, immune platelet purpura, and liver function damage than alemtuzumab in terms of severe adverse reaction. A previous study stressed that natalizumab had obvious decrease in the rate of relapse and the progression of the disease, which indicating that the adverse reactions and immunogenicity of natalizumab should be considered in the treatment of MS.^[29]^ Another study also emphasized on the serious adverse reactions of natalizumab, such as hepatotoxity, melanoma, headache, fatigue, hypersensitivity reactions, and arthralgia, which should be considered during the treatment of MS.^[30]^ What is more, several prior studies illustrated that autoimmune adverse events (AEs) represented a significant risk related to alemtuzumab therapy due to the repopulation of lymphocyte, and one of the obvious AEs was the increase in autoantibodies and the development of idiopathic thrombocytopenic purpura.^[31–33]^ Regardless, the role of individual treatment and the incidence of AEs were still unpredictable,^[34]^ hence, in this study, we analyzed and compared the adverse reactions of natalizumab and alemtuzumab in the treatment of MS and found that natalizumab had lesser adverse reactions than alemtuzumab for MS.

## 5 Conclusion

To conclude, our evidence indicated that natalizumab had better efficacy and lower adverse reactions than alemtuzumab, the treatment of RRMS patients while no significant difference was observed among other stages of MS patients. It could provide theoretical basis and clinical guidance for the treatment of different stages of MS. However, due to the limitation of the number of therapies and sample size of the study, more research and profound investigations are needed to explore further advancements and to aid physicians treating MS.
